# Overall satisfaction of health care users with the quality of and access to health care services: a cross-sectional study in six Central and Eastern European countries

**DOI:** 10.1186/s12913-016-1585-1

**Published:** 2016-08-02

**Authors:** Tetiana Stepurko, Milena Pavlova, Wim Groot

**Affiliations:** 1 School of Public Health, National University of Kyiv-Mohyla Academy, Kyiv, Ukraine; 2 Department of Health Services Research, CAPHRI, Maastricht University Medical Center, Faculty of Health, Medicine and Life Sciences, Maastricht University, Maastricht, The Netherlands; 3 Topinstitute Evidence-Based Education Research (TIER), Maastricht University, Maastricht, The Netherlands

**Keywords:** Consumer satisfaction, Outpatient service, Hospitalization, Informal patient payments, Central and Eastern Europe

## Abstract

**Background:**

The measurement of consumer satisfaction is an essential part of the assessment of health care services in terms of service quality and health care system responsiveness. Studies across Europe have described various strategies health care users employ to secure services with good quality and quick access. In Central and Eastern European countries, such strategies also include informal payments to health care providers. This paper analyzes the satisfaction of health care users with the quality of and access to health care services. The study focuses on six Central and Eastern European countries (Bulgaria, Hungary, Lithuania, Poland, Romania and Ukraine).

**Methods:**

We use data on past experience with health care use collected in 2010 through uniform national surveys in these countries. Based on these data, we carry out a multi-country analysis to investigate factors associated with the satisfaction of health care users in the six countries.

**Results:**

The results indicate that about 10-14 % of the service users are not satisfied with the quality of, or access to health care services they used in the preceding year. However, significant differences across countries and services are observed, e.g. the highest level of dissatisfaction with access to outpatient services (16.4 %) is observed among patients in Lithuania, while in Poland, the level of dissatisfaction with quality of outpatient and inpatient services are much lower than dissatisfaction with access. The study also analyses the association of users’ satisfaction with factors such as making informal payments, inability to pay and relative importance of service attributes stated by the service users.

**Conclusions:**

These multi-country findings provide evidence for health policy making in the Central and Eastern European countries. Although the average rates of satisfactions per country are relatively high, the results suggest that there is ample room for improvements. Specifically, many service-users still report dissatisfaction especially those who pay informally and those unable to pay. The high shares of informal payments and inability of users to deal with the health expenditures lead to doubts about the fairness of the health care provision in Central and Eastern Europe. There is an urgent need for policy makers in the region to not only acknowledge but also to effectively address this key problem.

**Electronic supplementary material:**

The online version of this article (doi:10.1186/s12913-016-1585-1) contains supplementary material, which is available to authorized users.

## Background

The measurement of consumer satisfaction is an essential part of the assessment of health care services in terms of service quality and health care system responsiveness [1, 2]. The interest in consumer satisfaction has become especially noticeable since the role of the patients in the health care system was reconsidered. The view of passive and uniformed patients is now replaced by the view of health care consumers, who are (or should be) actively involved in the medical decision-making process and in the achievement of good health outcomes [3–5]. As a response to this, numerous studies on consumer expectations, preferences and consumer choices have been published [3, 4, 6–9]. Some authors even argue that it is mostly the clients’ values and expectations that matter for the assessment of providers’ performance ([10] cited in [4]). As stated by Hekkert et al. ([3], p.68), satisfied patients ‘are more likely to continue using health care services, comply with medical treatment, maintain the relationship with a specific health care provider and recommend the health care service to others’.

Following from the above, it is not surprising that consumer satisfaction is often used interchangeably with patient satisfaction although some conceptual differences between health care consumers and patients are still noted [11]. Irrespective of terminology used however, the concept of satisfaction (either consumer or patient satisfaction) has several weaknesses, which are widely discussed in the literature, for example its subjectivity [3, 12] and the lack of unified understanding [13–15]. As Williams ([4], p.509) describes, ‘the number of assumptions are often made concerning what patients actually mean when they say they are satisfied’. In order to address this criticism, consumer satisfaction is often studied through actual patients’ experience with service utilization [1, 12]. Indeed, national and international health consumer indices present both health outcomes and patients’ experience and perceptions (including consumer satisfaction) in the assessment of health care systems and services [16, 17]. These health consumer indices suggest a recognition and legitimization of the concept of consumer satisfaction in terms of both satisfaction with the health care system and satisfaction with the health care services used.

This paper focuses on the consumer satisfaction with the use of health care services in Central and Eastern European countries. It contributes to the literature as consumer satisfaction data are sparse and not very frequently exploited in this European region [18, 19]. Available data on the satisfaction with the health care systems in Central and Eastern Europe, suggest that this aspect of consumer satisfaction is rather low compared to that in Western Europe. In fact, surveys of Gallup International and Gallup World Poll show that the poorest post-communist countries (e.g. Ukraine) are most dissatisfied with their lives, as well as with their health and their health care system [20]. At the same time, national studies have reported on various strategies the users of health care services in Central and Eastern Europe employ to secure services with good quality and quick access (including informal payments to health care providers and use of personal connections) [19, 21, 22]. It is however unclear to what extent they achieve this objective: what is the level of their satisfaction with the services used and how does this differ across the countries and consumer groups? This is the key question in this paper.

Specifically, this paper aims to analyze the satisfaction of health care users with the quality of and access to health care services in six Central and Eastern European countries (Bulgaria, Hungary, Lithuania, Poland, Romania and Ukraine). Based on nationally representative data collected in 2010 through uniform surveys in these countries, we perform a multi-country analysis to investigate cross-country differences and factors associated with the satisfaction of health care users in the six countries. We are interested in Central and Eastern European countries because they experienced a similar development of health care reforms that shifted their Semashko health care systems established during the communist era, towards social health insurance [23]. Ukraine is the only one among the countries in our study where social health insurance is not yet introduced. Another distinctive feature is that it is a non-EU country. By and large, during the post-communist time, these countries experienced comparable but still different challenges in quality of governance, public service provision, informal economies, and health care reforms [23]. These similarities and diversities provide a base for insightful cross-country comparisons.

### Health care satisfaction studies in Central and Eastern Europe

Generosity in public health care services financing is rarely observed in the health care systems of Central and Eastern Europe [24]. Public health care resources remain insufficient to maintain the large health care infrastructure established during the communist period. At the same time, the available resources are allocated and used inefficiently, which further aggravates the health care financial problems [25, 26]. Moreover, system transparency and good governance have been and remain challenging issues in Central and Eastern Europe. These aspects of health care are closely linked to the satisfaction with the health care system measured in surveys. In the early 1900s, the satisfaction with the public health care system in Central and Eastern Europe dropped primarily due to the increase of private expenditure and out-of-pocket payments [27]. At the same time, informal cash payments to health care providers became common [28–30]. Informal patient payments, especially when combined with formal charges, constitute a major barrier to health care access but also an incentive for better health care provision [31, 32]. This in turn reflects on the satisfaction with the health care system in general and health care services in particular. Indeed, a higher share of private expenditures and the need to pay informally may result in reduced consumer satisfaction and low patients’ trust in the health care system [27, 33].

Previous studies have provided some evidence on the linkage between informal payment and consumer satisfaction with health care services. For example, some consumers of government health care services in the Baltic region describe positive effects of unofficial payment, namely ‘a personal sense of satisfaction’ as well as better access and quality of service [34]. As Gaal et al. [31] suggest, ‘quality-conscious consumers may try to “buy” better services with informal payments’. The relation between informal payments and consumer satisfaction has been studied outside the Central and Eastern European region as well. In Greece, for example, the results of a quantitative study demonstrate that the size of the extra payment does not have a significant association with the level of satisfaction [35]. At the same time, a cross-country comparison in Europe and Central Asia has found that the patients’ perceived the need to make unofficial payments has a significant association with the level of dissatisfaction with the services received [19].

Taking into account the few studies on factors associated with consumer satisfaction with health care services, particularly in Central and Eastern Europe, as well as the inconsistency in findings reported, our study focuses on these issues. We are aware of the fact that a variety of methodological guidelines in the area of satisfaction measurement are developed [36–38]. The most recent studies operate with composite satisfaction ratings on perceived quality, access and effectiveness of care received [39]. Ware et al. [40] define eight dimensions of patient satisfaction that have been addressed in published studies: art of care (for example, personal qualities), technical quality of care (related to provider competence), accessibility or convenience, finances, physical environment, availability, continuity and efficacy or outcomes of care. However, we focus only on the access and quality dimensions of the satisfaction of health care users with services. Thus, no pretense is made that all consumer or patient satisfaction aspects are covered in this study.

Previous studies have reported three key individual determinants of satisfaction: expectations, health status and socio-demographic characteristics [41]. Stable associations of patient satisfaction with age, health status and education are often identified while the gender indicator does not show consistent linkage with satisfaction [3, 42]. Apart from socio-demographic features of the patients, Hekkert et al. [3] provide empirical evidence, which suggests that a ‘minor part of the variance in patient satisfaction scores is attributed to the hospital and department levels’ rather than age, health status and education which are the most important determinants of patient satisfaction. Beich et al. [1] identify the type of care and immunization coverage together with previously mentioned aspects as significant predictors of patient satisfaction with the health care system. However, the major impact on satisfaction is attributed to broader societal factors. Also, it is reported that inpatient service users demonstrate higher levels of satisfaction compared to outpatient service users [1]. In this study, we compare the satisfaction of health care users with outpatient services and inpatient services. We also study whether the level of satisfaction is associated with socio-demographic factors, country of residence, experience of paying informally, inability to pay for health care service and the importance that consumers attach to service attributes. A study on the joint association of these factors with the satisfaction of health care users has not been previously reported.

## Methods

This paper uses the dataset obtained through the multi-country quantitative survey entitled ‘Willingness and ability of patients to pay for medical services’ that was conducted within the FP7 project “Assessment of patient payment policies and projection of their efficiency, equity and quality effects. The case of Central and Eastern Europe” (project website: www.assprocee2007.com). The project, as well as the survey, was co-funded by the European Commission under FP7 Theme 8 Socio-economic Sciences and Humanities (GA no. 217431). The survey was in line with the project objectives, namely analysis of the pattern of formal and informal payments in European countries.

Six Central and Eastern European countries, namely Bulgaria, Hungary, Lithuania, Poland, Romania, and Ukraine, were included in this multi-country survey. Thus, the survey comprised six uniform national representative surveys conducted during the summer of 2010 (one national survey per country).

The coordinator of the project (Maastricht University) guided the development of the survey questionnaire (the research instrument) by the project partners. The parts of the initial questionnaire draft were pre-tested in different countries. For example, the initial pre-test of the informal payments part of the questionnaire was conducted in Ukraine using the experience of eight respondents with different socio-demographic profiles. First, we checked the appropriateness of the mode of data collection and it appeared that self-administrated mode (suggested for sensitive topics) could not work well with older people. Still, informal patient payments appeared to be rather non-sensitive topic as respondents shared their experience openly and did not look confused with the questions. Also, we checked whether our terminology is understood by non-experts and as a result, in addition to informal payments, we added “cash payments and in-kind gifts” that is more commonly used by the patients. We also asked the respondents in the initial pre-test to answer the questions and to reflect on the difficulties in understanding the wording of the questions. After the initial pre-tests, we changed the format of the questions and we decreased the number of the questions. Meanwhile, in order to improve the questionnaire, we turned to external experts in the field to review the questions and advise adjustments.

After the compilation of the entire questionnaire in English, following Brislin’s [43] suggestions for a cross-country instrument development, the questionnaire was translated in local languages and verified using the backward translation method in order to assure consistency of the instrument and wording of the questions as well as to assure a meaningful comparison of the results between the countries. Before the backward translation however, the questionnaire was pre-tested in the six countries via face-to-face interviews with 30 respondents with different social-demographic characteristics (convenient sample). Based on these country-level pre-tests, the wording of the questions in local languages was adjusted. The backward translation into English helped us to determine if the adjustments in local languages led to changes in the meaning and what additional adjustments were necessary. Thus, the standardized questionnaire (the research instrument) used in the multi-country survey was identical for all six countries.

The coordinator of the project (Maastricht University) ordered the data collection at Gallup International. Gallup International is an umbrella organization of independent research organizations in 70 countries and it has substantial experience with consumer studies including studies among health care consumers. This helped to assure the quality of the data collection, identical methodologies and comparative nature of the data across the countries. Thus, the development of the survey methodology (sampling method and questionnaire) was the end responsibility of a single project partner (the coordinator) and the execution of the data collection was the end responsibility of a single party (Gallup International). Gallup International adhered to the conditions of sampling and data collection defined by the project consortium in a contractual agreement and used the questionnaire as developed by the project team. Thus, the data collection was executed by Gallup International’s country representatives based on an identical methodology.

In line with this, for the empirical data collection, a nationally representative sample of the adult general population (18+ years) was drawn in each country following a multi-staged random probability approach. The data were collected in the same way in all countries through face-to-face interviews to reduce the chance of incorrectly filled in answers. In particular, in the beginning, approximately 100–150 sampling points per country were chosen in consideration of regional, urban/rural and ethnic characteristics; then, about 10 addresses/households per sampling point were selected using the random route method. To select addresses/households of potential respondents, the random route method was used, i.e. a starting point and direction were determined, the household selected for the survey was every forth address on the left and side of the street in urban areas, turning left at intersections and, after reaching a dead end, going back to the last crossing and further proceeding at random etc. In rural areas, every fourth inhabitable house on both sides of the interviewer’s route was selected. For the survey, interviewers selected one adult household member (18-years or older) using the “last birthday” principle, i.e. the interviewer asked to speak to the adult member of the household who had the last birthday. The last-birthday method is based on the assumption that the assignment of birthdates is a random process and also every household member has an equal chance of being selected [44]. Only one individual per household was interviewed. If the respondent determined on stage 3 refused or was unavailable to take part in an interview after two call backs recorded in the fieldwork report, a replacing respondent was identified following the same procedure. As a result, about 1000 respondents per country participated in the survey. The national surveys were conducted simultaneously in all six countries.

During the interviews conducted at the respondents’ home, trained interviewers used the standardized questionnaire which was designed to reveal past payments and experience with health care use, as well as preferences and willingness to pay for health care services. The average number of interviews per interviewer were kept limited (about 25) to reduce the interviewer bias. The average duration of one interview was about 30 min. Prior to an interview, the respondent was asked to read and sign an informed consent form. According to the national regulations in the countries where the surveys were carried out, an approval by ethical committee was not required for survey-based studies.

In this paper, we analyze the data on the satisfaction of health care users, socio-demographic characteristics, the importance attached to health care attributes, informal payments and inability to pay for the health care services used during the preceding 12 month. The wording of the questions used in this study is shown in Additional file 1, and the coding of the variables is presented in the tables where relevant. The central concepts of this paper, i.e. the satisfaction with access and satisfaction with quality, are presented to the health care users-respondents in a general way without detailed transcriptions because of (a) survey duration constraints and (b) cross-country differences in defining service priorities and attributes. We consider health care consumers’ satisfaction as a user-reported outcome measure [2] and as it is mentioned above there is a high degree of subjectivity in the satisfaction concept as well as in satisfaction with access and quality. If the respondent confirmed to be satisfied with either quality or access, we interpret this as a positive subjective assessment of the services that may influence future health care consumption behavior [45].

For the analysis of satisfaction with outpatient services, we extract a sub-sample of respondents who have used outpatient services during the preceding 12 months out of the nationally representative sample (users and non-users). Analogously, for the analysis of satisfaction with inpatient services, we use a sub-sample of respondents who have used inpatient services during the preceding 12 months. Thus, all analyses are performed using the corresponding sub-sample of users. We first analyze the data using descriptive statistics, then we run a regression analysis. Specifically, ordered probit regression analysis is applied to investigate the association between the level of satisfaction with quality of and access to outpatient and inpatient service (four dependent variables; range from 0 (dissatisfied with either quality or access) to 2 (satisfied) and the four groups of independent variables:

1. country indicators;
2. Individual health status as well as individual and household socio-demographic characteristics (presented in Additional file 2);
3. the experience with informal payments and whether the respondent needed to borrow funds to cover the treatment (need to take or borrow funds from family of friends, sell assets);
4. the self-explicated importance of six attributes of health care services ranked by each respondent: medical equipment and reputation/skills of the physician (clinical quality), condition of the facility (social quality), attitude of the staff (psychological access), travel time and waiting time (spatial and temporal access). The variables range from 1 – the highest importance to 7 – the least important).

The last group of independent variables is included to indicate personal expectations of the individual respondents. Respondents who attach a higher importance to a certain attribute would have higher expectations related to this attribute and would be more inclined to state dissatisfaction when the performance related to this attribute is lower. In contrast, respondents who attach a lower importance to that attribute might do not feel dissatisfied even though the performance related to this attribute is lower. We find it necessary to account for this aspect in our regression analysis, based on the data we have, because expectations are suggested as one of the key satisfaction determinant [41]. The distinction between quality-related and access-related attributes is made based on the theoretical framework of Berki and Ashcraft [46]. We include the quality-related attributes in the regression analyses on satisfaction with service quality and the access-related attributes in the regression analyses related to access satisfaction. Regression models for each country are also run in order to check the consistency of direction of the associations (these results are not presented since no essential differences are observed). The correlation between the independent variables included in the regression analysis is weak (absolute value of the correlation coefficient < .6) or insignificant (*p* > .05).

## Results

The response rate varies between the countries from the lowest in Poland and Ukraine (38 % and 42 % respectively) to the highest in Bulgaria and in Hungary (67 % and 76 % respectively). For Romania and Lithuania, the response rate is 56 % and 52 % respectively. The initial analysis of the country samples indicates that the sample characteristics related to age, gender, place of residence and household income, are comparable to the countries’ national statistics.

### Satisfaction with the quality and access to health care services

The overall pattern of service satisfaction suggests that about 10-14 % of the service users are not satisfied with the quality of or access to health care service they used (see Table 1). Still, we observe variations across countries as well as across services. In Lithuania, the share of users unsatisfied with service quality is about 6.1-8.5 % while the highest value is observed for dissatisfaction with access to outpatient services (16.4 %). Also, the satisfaction pattern shows a great variability in Poland: 9.2 % and 12.6 % of service users are not satisfied with the quality of outpatient and inpatient services respectively in contrast to 18.3 % and 20.3 % who are dissatisfied with access to such services. When the share of service consumers who report satisfaction is compared among the six countries, Hungarian service users on average are more satisfied with quality and access (67.3 %–70.3 %) of the services used than service users in Poland (39.6 %–51.0 %) and Ukraine (41.4 %–45.9 %).

**Table 1:** Satisfaction of health care users with the quality of and access to health care services used during the preceding 12 months^a^

		Bulgaria		Hungary		Lithuania		Poland		Romania		Ukrainian		Total	
Outpatient services	Sample size	*N* = 736		*N* =826		*N* =739		*N* =735		*N* =651		*N* =572		*N* =4259	
		*n*	%	*n*	%	*n*	%	*n*	%	*n*	%	*n*	%	*n*	%
Are you overall satisfied with the quality of outpatient physician services that you used during last 12 months?	No	120	16.8	38	4.6	61	8.4	67	9.2	81	12.8	82	14.5	449	10.7
	Somewhat	267	37.3	207	25.1	266	36.4	288	39.7	156	24.6	249	44.1	1433	34.2
	Yes	329	45.9	580	70.3	403	55.2	370	51.0	398	62.7	234	41.4	2314	55.1
Are you overall satisfied with the access to outpatient physician services that you used during last 12 months?	No	124	17.5	53	6.4	120	16.4	132	18.3	76	11.9	101	17.8	606	14.5
	Somewhat	240	33.8	207	25.1	231	31.6	303	42.1	161	25.3	230	40.6	1372	32.7
	Yes	346	48.7	566	68.5	380	52.0	285	39.6	400	62.8	236	41.6	2213	52.8
Inpatient services	Sample size	*N* = 171		*N* =219		*N* =165		*N* =159		*N* =192		*N* =184		*N* =1090	
		*n*	%	*n*	%	*n*	%	*n*	%	*n*	%	*n*	%	*n*	%
Are you overall satisfied with the quality of inpatient hospital services that you used during last 12 months?	No	25	15.0	15	6.9	10	6.1	20	12.6	23	12.2	27	14.8	120	11.1
	Somewhat	51	30.5	56	25.8	59	36.0	63	39.6	75	39.7	75	41.2	379	35.2
	Yes	91	54.5	146	67.3	95	57.9	76	47.8	91	48.1	80	44.0	579	53.7
Are you overall satisfied with the access to inpatient hospital services that you used during last 12 months?	No	22	13.2	14	6.5	14	8.5	32	20.3	26	13.8	21	11.5	129	12.0
	Somewhat	50	29.9	55	25.3	53	32.3	59	37.3	65	34.4	78	42.6	360	33.4
	Yes	95	56.9	148	68.2	97	59.1	67	42.4	98	51.9	84	45.9	589	54.6

^**a**^Differences between N and the sum of n per question are due to missings

### Informal payments and inability to pay for health care services

Table 2 shows the valid percentage and number of service users per country who paid for health care services within the preceding 12 months. On average, about 22 % of outpatient service users paid informally and about 12 % of payers report being unable to pay for such services. The highest share of service users who report informal payments for outpatient services is in Ukraine (37.4 %) and in Romania (34.6 %). In these countries, inability to pay for outpatient services is also high (22.4 % in Romania and 18.6 % in Ukraine). Much lower shares of informal payers and service users unable to cover the related out-of-pocket expenditures are observed in Poland (5.9 % and 13.2 % respectively) and in Bulgaria (12.4 % and 5.7 % consequently). Still, in Hungary, we observe a quite substantial share of outpatient service users who pay informally (21.9 %), however only 5.6 % of them report to be unable to pay.

**Table 2:** Informal patient payments and inability to pay for health care services reported by service users (only respondents who have used the specified services during the preceding 12 months)^a^

		Bulgaria		Hungary		Lithuania		Poland		Romania		Ukrainian		Total	
Outpatient services	Sample size	*N* = 736		*N* =826		*N* =739		*N* =735		*N* =651		*N* =572		*N* =4259	
		*n*	%	*n*	%	*n*	%	*n*	%	*n*	%	*n*	%	*n*	%
Informal payments for outpatient physician services	No	645	87.6	645	78.1	584	79.0	676	92.0	426	65.4	358	62.6	3334	78.3
	Yes	91	12.4	181	21.9	155	21.0	59	5.9	225	34.6	214	37.4	925	21.7
Outpatient physician services: Was it necessary to take or borrow cash from family, friends?	No	525	94.3	221	94.4	281	98.9	158	86.8	288	77.6	263	81.4	1736	87.5
	Yes	32	5.7	13	5.6	35	11.1	24	13.2	83	22.4	60	18.6	247	12.5
Inpatient services	Sample size	*N* = 171		*N* =219		*N* =165		*N* =159		*N* =192		*N* =184		*N* =1090	
		*n*	%	*n*	%	*n*	%	*n*	%	*n*	%	*n*	%	*n*	%
Informal payments for inpatient hospital services	No	126	73.7	118	53.9	79	47.9	130	81.8	83	43.2	87	47.3	623	57.2
	Yes	45	26.3	101	46.1	86	52.1	29	18.2	109	56.8	97	52.7	467	42.8
Inpatient hospital services: Was it necessary to take or borrow cash from family, friends?	No	94	81.0	91	88.3	74	76.3	27	75.0	77	63.1	76	56.7	439	72.2
	Yes	22	19.0	12	11.7	23	23.7	9	25.0	45	36.9	58	44.3	169	27.8

^**a**^Differences between N and the sum of n per question are due to missings

Concerning inpatient hospital services during the preceding 12 months, about 43 % of the service users in the six countries paid informally and 27.8 % of the users report that they had to borrow money in order to cover the expenditures. Slightly more than half of inpatient service users pay informally for hospitalization in Lithuania (52.1 %), Ukraine (52.7 %) and Romania (56.8 %). However, 36.9 % of inpatient service users in Romania and 44.3 % in Ukraine were unable to pay for hospitalization in contrast to 23.7 % in Lithuania. In Bulgaria, there is virtually the same share of those who have to take or borrow funds (19 %), however the share of informal payers for hospitalization is lower (26.3 %). Poland has the lowest share of inpatient service users who pay informally and moderate level of service users, who are unable to pay for hospitalizations. Hungary has quite a high share of service users who pay informally for inpatient care (46.1 %). However, inability to pay is only minor (11.7 %), which corresponds to the outpatient pattern.

### Importance of health care service attributes

Table 3 presents the rank order of importance which the users of given services attach to the attributes of these services. In particular, the table shows the mean and median values per country and in total. For both outpatient physician services and for inpatient hospital services, the service users attach the highest importance to ‘the reputation and skills of physician/surgeon’ while the relatively least important attribute appears to be ‘travel time’. For outpatient physician services, the second (median value 3.0) and the third (median value 4.0) ranks of attribute importance are given to ‘medical equipment’ and ‘attitude of the staff’ respectively. Such ordering is also observed for inpatient hospital services, but the value of the median is 2.0 and 4.0 respectively.

**Table 3:** Importance of health care services attributes ranked by service users (only respondents who have used the specified services during the preceding 12 months)

Each attribute is given a rank from 1- the most important to 7 – the least important		Bulgaria	Hungary	Lithuania	Poland	Romania	Ukrainian	Total
OUTPATIENT PHYSICIAN SERVICES		*N* = 736	*N* =826	*N* =739	*N* =735	*N* =651	*N* =572	*N* =4259
Medical equipment in the physician office	Median	3.0	3.0	2.0	3.0	3.0	3.0	3.0
	Mean (St.deviat.)	2.94 (1.42)	3.03 (1.49)	273 (1.34)	3.32 (1.60)	3.05 (1.60)	2.91 (1.33)	3.0 (1.48)
Reputation and skills of physician	Median	1.0	1.0	1.0	1.0	1.0	1.0	1.0
	Mean (St.deviat.)	1.52 (0.93)	2.01 (1.48)	1.51 (1.01)	1.89 (1.53)	2.01 (1.55)	1.68 (1.26)	1.8 (1.33)
Condition of the physician office	Median	4.0	5.0	5.0	5.0	4.0	5.0	5.0
	Mean (St.deviat.)	4.24 (1.44)	4.68 (1.65)	5.16 (1.48)	4.44 (1.60)	4.00 (1.44)	5.01 (1.42)	4.59 (1.56)
Attitude of the staff	Median	4.0	3.0	3.0	4.0	4.0	3.0	4.0
	Mean (St.deviat.)	3.63 (1.50)	3.37 (1.59)	3.53 (1.40)	4.01 (1.69)	3.58 (1.51)	3.47 (1.43)	3.6 (1.5)
Travel time to the physician’s office	Median	6.0	6.0	6.0	6.0	6.0	6.0	6.0
	Mean (St.deviat.)	5.86 (1.45)	5.46 (1.65)	5.71 (1.43)	5.39 (1.67)	5.86 (1.59)	5.73 (1.49)	5.7 (1.56)
Waiting time in front of the physician office	Median	6.0	5.0	5.0	5.0	6.0	6.0	5.0
	Mean (St.deviat.)	5.44 (1.44)	4.61 (1.69)	4.62 (1.57)	4.54 (1.73)	5.15 (1.61)	5.37 (1.44)	4.96 (1.63)
Amount of money paid by the patient	Median	5.0	5.0	5.0	5.0	5.0	4.0	5.0
	Mean (St.deviat.)	4.36 (1.88)	4.84 (1.95)	4.73 (1.79)	4.40 (2.12)	4.36 (1.91)	3.83 (1.88)	4.42 (1.95)
INPATIENT HOSPITAL SERVICES		*N* = 171	*N* =219	*N* =165	*N* =159	*N* =192	*N* =184	*N* =1090
Medical equipment at the hospital	Median	2.0	2.0	2.0	3.0	2.0	2.0	2.0
	Mean (St.deviat.)	2.72 (1.33)	2.78 (1.39)	2.41 (1.18)	3.10 (1.54)	2.90 (1.56)	2.71 (1.22)	2.77 (1.39)
Reputation and skills of the surgeon	Median	1.0	1.0	1.0	1.0	1.0	1.0	1.0
	Mean (St.deviat.)	1.49 (0.93)	1.93 (1.38)	1.45 (0.83)	2.00 (1.50)	1.95 (1.52)	1.65 (1.14)	1.75 (1.26)
Condition of the hospital interior	Median	4.0	4.0	5.0	4.0	4.0	5.0	5.0
	Mean (St.deviat.)	3.89 (1.30)	4.92 (1.54)	5.04 (1.42)	4.41 (1.52)	3.92 (1.47)	4.86 (1.41)	4.51 (1.52)
Attitude of the staff	Median	4.0	4.0	4.0	4.0	4.0	4.0	4.0
	Mean (St.deviat.)	3.99 (1.42)	3.70 (1.51)	3.78 (1.34)	4.19 (1.67)	3.84 (1.43)	3.67 (1.39)	3.86 (1.47)
Travel time to the hospital	Median	7.0	6.0	7.0	6.0	7.0	6.5	7.0
	Mean (St.deviat.)	6.26 (1.21)	5.87 (1.49)	6.24 (1.15)	5.76 (1.58)	5.98 (1.57)	6.04 (1.36)	6.02 (1.41)
Waiting time for the operation	Median	6.0	4.0	5.0	4.0	5.0	6.0	5.0
	Mean (St.deviat.)	5.32 (1.35)	4.04 (1.72)	4.50 (1.32)	4.06 (1.80)	5.09 (1.54)	5.30 (1.41)	4.71 (1.63)
Amount of money paid by the patient	Median	5.0	5.0	5.0	5.0	5.0	4.0	5.0
	Mean (St.deviat.)	4.33 (1.85)	4.77 (1.88)	4.58 (1.82)	4.48 (2.06)	4.32 (1.89)	3.77 (1.88)	4.38 (1.92)

For the cross-country pattern of the importance ranking of service attributes, we do not observe variation of the median for the following attributes: reputation and skills of the provider (median 1.0), travel time to the physician’s office (6.0) and attitude of the hospital staff (4.0 median). For the other attributes, some differences are observed, e.g. in Lithuania for physician services, medical equipment seems to be a more important attribute to service users compared to other countries (median is 2.0 for Lithuania and 3.0 for others), while in Poland for hospitalization, service users attach lesser importance to equipment (median is 3.0 in Poland and 2.0 in other countries). ‘Amount of money paid by the patient’ is ranked as a more important attribute by service users in Ukraine (4.0 median) compared to other countries (5.0 median). ‘Condition of physician office’ is valued more by service users in Bulgaria and Romania (4.0 median) compared to the other four countries, and ‘condition of hospital interior’ is ranked lower in Lithuania and Ukraine (5.0 in contrast to others’ 4.0 median).

### Results of regression analyses

Table 4 presents the results of four ordered probit regression analyses. The regression results show that in all four models, the experience of making informal payments as well as the inability to pay for health care services have a negative significant association with the satisfaction of health care users regarding both quality of and access to health care service. In particular, those who pay informally or are forced to borrow money or sell assets, are also more likely to be unsatisfied with the services used.

**Table 4:** Ordered probit regression analysis on the satisfaction of health care users with the quality of and access to health care services used during the preceding 12 months

Data collection year: 2010	Outpatient physician services		Inpatient hospital services	
	Quality satisfaction	Access satisfaction	Quality satisfaction	Access satisfaction
	[0 - No, 1 - Somewhat, 2 - Yes]			
	Coefficient (S.E.)	Coefficient (S.E.)	Coefficient (S.E.)	Coefficient (S.E.)
Paid informally for the service [0-No; 1-Yes]]	−.635* (.082)	−.591* (.081)	−.373* (.143)	−.384* (.143)
Was necessary to borrow [0-No; 1-Yes]	−.706* (.138)	−.489* (.136)	−.478* (.183)	−.538* (.182)
Medical equipment – *indicator on quality importance* [from 1 – the most important to 7]	.041** (.023)	–	−.025 (.047)	–
Reputation and skills – *indicator on quality importance* [from 1 – the most important to 7]	−.038 (.026)	–	−.037 (.052)	–
Condition of the facility - – *indicator on quality importance* [from 1 – the most important to 7]	−.048* (0.22)	–	−.025 (.045)	–
Attitude of the staff – *indicator on access importance* [from 1 – the most important to 7]	–	−.030 (.021)	–	−.110* (.045)
Travel time - *indicator on access importance* [from 1 – the most important to 7]	–	.068* (.020)	–	−.006 (.047)
Waiting time - *indicator on access importance* [from 1 – the most important to 7]	–	.107* (.020)	–	.087* (.043)
Bulgaria [0-No; 1-Yes]	−.654* (.112)	−.194** (.109)	−.510* (.251)	−.359 (.244)
Hungary [0-No; 1-Yes]	.625* (.115)	.740* (.111)	.370 (.238)	.296 (.238)
Poland [0-No; 1-Yes]	−.341* (.114)	−.508* (.110)	−.752* (.251)	−1.081* (.249)
Romania [0-No; 1-Yes]	.207** (.137)	−.580* (.115)	−.394** (.239)	−.252 (.229)
Ukraine [0-No; 1-Yes]	−.472* (.122)	−.172 (.121)	−.598* (.234)	−.451** (.237)
Age [Years]	.007* (.002)	.003 (.002)	.013* (.004)	.011* (.005)
Gender [0 - Male; 1 - Female]	.018 (.066)	.013 (.065)	−.122 (.129)	−.197 (.131)
Residence place [0 – Town or city; 1-Village]	.112 (.072)	.022 (.071)	.128 (.139)	−.079 (.140)
Level of education [From 0 - Uncompleted primary education to 5 - Tertiary education]	−.064* (.032)	−.055** (.032)	−.004 (.064)	−.042 (.065)
Major health problems confirmed by a physician [0 - No; 1 - Yes]	−.191* (.075)	−.116 (.074)	−.282* (.160)	−.212 (.162)
Number of people in the household [Number]	−.029 (.029)	−.039 (.029)	−.016 (.058)	.034 (.059)
Net average household income per month [18 categories, from the lowest to the highest]	−.020** (.012)	−.008 (.012)	−.015 (.025)	−.005 (.025)
Treshold = 0	−2.865* (.264)	−1.570* (.281)	−2.762* (.550)	−2.492* (.610)
Treshold = 1	−.810* (.259)	.223 (.280)	−.651 (.541)	−.497 (.603)
Number of observations	3910	3908	999	1000
Nagelkerke R Square	.096	.099	.096	.105

*p < 0.05; **p ≤ 0.10

Are you overall satisfied with the QUALITY of physician services that you used during the last 12 months - yes, no, somewhat?

Are you overall satisfied with the ACCESS to physician services that you used during the last 12 months - yes, no, somewhat?

Regarding the importance of service attributes, it appears that there are significant associations in the outpatient model only. Indeed, we observe a higher probability of being satisfied with quality of outpatient services for health care users who attach lower importance to medical equipment as well as for those who attach higher importance to the condition of the facility. Among the access-related attributes of the services, the importance of travel time and waiting time have a positive significant association with the satisfaction with access to outpatient services. The importance of waiting time also has a positive association in the inpatient model, while ‘attitude of the staff’ indicator correlates negatively with hospitalization satisfaction. Those who attach higher importance to staff attitude (psychological access) are less satisfied with the overall access to hospital services.

We observe negative significant coefficients for the country indicator ‘Poland’ in all four regression analyses suggesting that Polish health care users are less satisfied with health care services they use than those in Lithuania. The opposite is observed in Hungary where outpatient service users are more satisfied with care than those in Lithuania. We also observe a positive significant association between the users’ satisfaction with quality of outpatient service in Romania. The country indicators for Bulgaria and Ukraine are negative in three outpatient and four inpatient models (i.e. not in the model of satisfaction with outpatient access for Ukraine, and with access to hospitalization for Bulgaria).

Older health care users have a higher probability of being satisfied with health care service. Health care users, who have major health problems confirmed by a physician are more frequently dissatisfied with quality of services, while respondents with a higher education are mostly dissatisfied with outpatient services (both quality and access). In addition, household income has a negative significant correlation in the satisfaction model on quality of outpatient service. Residence place and gender do not correlate significantly with the dependent variables.

As mentioned in the Methods section, we run the four regression models per country but we do not observe any notable differences that change the key finding reported above.

## Discussion

In our cross-sectional study, we have looked at the satisfaction of health care users with (a) access to and (b) quality of two groups of health care services (out- and inpatient ones) in six Central and Eastern European countries. We also looked at the association between the satisfaction of health care and four groups of factors: socio-demographic characteristics, health status, informal payments, inability to pay for health care services, and the self-explicated importance of service attributes as an indicator of personal expectations.

Our findings indicate satisfaction patterns similar to previous studies. In particular, the significant associations that we find between the satisfaction of health care users and some socio-demographic characteristics (age, level of education and presence of health problem) are supported by previous studies [19, 47]. People with a bad health status who are more in need of and have experience with service use are typically less satisfied [3, 47–49]. Hall et al. [48] emphasize that the linkage between satisfaction and health status is least discussed because of the complexity of the statistical grounds for the analysis of this relation, and because of the different possible interpretations. Indeed, interconnectivity between health status and satisfaction is underlined, namely those with worse health status are also prone to be less satisfied ([50, 51] cited in [48]). It should be noted however, that the nature of the health problem, its measurement and type of analysis also matter [52]. The second possible interpretation concerns the lack of a system response to the needs of the sick person who becomes distressed and thus ‘the distressed individuals may unwittingly produce negative behavior in providers’ ([53] cited in [48]). Still, the management of health care problems is an essential layer in the service provision system and it should include improving service continuity, trustful patient-provider relations, adequate length and manner of communication, etc. [54].

In addition to the findings supported by previous studies, our analysis also provides new insights into cross-country differences. For example, health care users in Hungary report relatively low levels of dissatisfaction with the services they used compared to the other countries. As the Hungarian health care system ranks highest in the Euro Health consumer index among the countries included in our study [24], this higher level of satisfaction is to be expected. At the same time, the health care systems of Poland, Lithuania, Ukraine and Bulgaria are rather different but our study reveals a common low level of satisfaction with access to outpatient services. About one fifth of outpatient service users in the countries are dissatisfied with the access to these services. This is especially noticeable in Lithuania where 16.4 % of outpatient services users are dissatisfied with the access to outpatient care, while only 6.1–8.5 % report dissatisfaction with other dimensions of satisfaction measured in our study. Moreover, in Bulgaria and Ukraine, we observe dissatisfaction with both quality and access to outpatient care. Previous studies also cast doubt on the service quality in these two countries [55–60]. Although most of Central and Eastern European countries focused their health care reforms on strengthening primary care (introducing GPs or family doctors), low GPs job satisfaction [61] and ‘wasted’ time of patient and physician because of restricted access to specialists [62] are reported. Indeed, the gate-keeping role of GPs creates dissatisfaction among the outpatient services users as people in these countries are used to and often prefer direct access to more specialized care. Zielinski et al. [63] argue that ‘patients who are accustomed to easy access to specialist treatment in large polyclinics, especially in cities, may find it difficult to accept a new primary health care system in which the family physician is the main, and often only, caregiver.’ It is one of the possible interpretations of the higher satisfaction rate of Hungarian outpatient service users, where the system is based on a partial GP gate-keeping role in contrast for example to the Lithuanian primary health care system, which has a GP gate-keeping role [64].

Further, more detailed studies that have specifically focused on the satisfaction of health care users can help to understand the variations within and across the countries. For example, the level of satisfaction with health care services can be related to prior expectations linked to the personal experience and knowledge of health care users [1, 2]. Thus, health care users with lower expectations, who lack knowledge or are passive and uncritical, might state higher satisfaction with services than those with higher expectations. Also, health care users who have chosen to pay informally might try to justify these payments by stating higher satisfaction. However, this would not necessarily apply to the cases when the informal payments are requested or indicated by the staff. To study these and other relevant aspects of consumer satisfaction with health care services more detailed national studies are needed.

Moreover, the level of satisfaction with the quality of and access to health care services measured in our study is relatively high. Against the background of other studies that report low consumer satisfaction with the health care system in the Central and Eastern European countries [20], our results may indicate that health care users in these countries employ personalized tools to secure health care services they desire. The use of personal connections (social network) is evidenced to be an effective approach in this direction [65]. Another approach is the use of informal payments to ‘bribe’ the physician to provide better and easily accessible services [66]. This however, does not necessary apply when such payments are directly requested by providers. In view of this, paying informally does not necessary mean obtaining better services. As suggested by our regression results, in fact, paying informally is associated with increased dissatisfaction with health care services. Thus, further studies are needed to explore how and to what extent the purpose and initiator of the informal payment affect consumer satisfaction. Such causal analysis should consider the possible double-sided relation between satisfaction and informal payments, i.e. paying informally may influence the satisfaction of health care users but also informal payments may be made due to low satisfaction with services.

However, the negative association between the satisfaction with the health care used and the inability to pay for care that we find across the countries is rather straightforward. It essentially confirms the assumptions of earlier studies [27, 33] on the negative linkage between satisfaction and out-of-pocket patient payments. The inability to pay levels observed in our study are rather high and especially pronounced in case of inpatient services. Thus, measures to avoid a catastrophic financial burden by patients and their families, as well to avoid the need of foregoing necessary medical care for those who cannot afford pay, require the urgent attention of policy makers in the region.

We can compare the above associations for the overall satisfaction with services used, to the associations reported in a previous EU study for the overall satisfaction with the health care in the country [67, 68]. This comparison shows that younger respondents and those with financial difficulties who according to our study, are less satisfied with the health care services they used, are also less satisfied with the health care in their country. However, for education, this comparison is the reverse. According to our study, those with higher education are less satisfied with the services they used while the results of the EU study show that higher educated individuals are more likely to give a higher rate for their satisfaction with the health care in their country. However, this discrepancy may well result from the fact that our study only includes Central and Eastern European countries and not all EU member states. Another explanation can be that the satisfaction with services used a user-based indicator, while the satisfaction with health care in the country is a citizen-based indicator.

It should be mentioned however, that the same EU study reports a higher importance attached to physician’s skills and medical equipment by citizens in Bulgaria, Hungary, Lithuania and Romania, compared to waiting time and proximity, which is in accordance with our findings. For Poland however, the EU study reports a higher importance attached to waiting time than to physician’s skill, which is different to our findings. The EU study also confirms a low satisfaction level with the health care in the country in general for all five EU member states included in our analysis compared to EU member states in Western Europe. This can be well explained by the analogous differences in the health care system performance across EU [69].

As mentioned at the outset of this paper, consumer satisfaction research has a number of (unavoidable) limitations, some of which apply to our study as well. The most noticeable challenges can be attributed to the measurement of satisfaction and its interpretation. These difficulties result from the complex nature of the satisfaction concept as satisfaction may have an idiosyncratic element and ‘may originate in factors outside of the health care system’ [4]. Although satisfaction as a patient perspective has been legitimized in service provision in order to extend the clinical outcomes angle [4], the satisfaction of health care consumers with health care services or systems does not necessarily indicate better performance [47]. A variety of external factors matter: differences in personal expectations, the country discourse and media image, as well as the construction of satisfaction as a public opinion. These limitations in satisfaction surveys are difficult to overcome, although we have taken some measures to minimize biases: the aspects of care (quality and access) have been explicitly stated in our questions as well as the importance of service attributes is included in the analysis as a proxy for personal expectations. As indicated by our regression results, the importance attached to service attributes, and thus personal expectations has a significant association with the satisfaction of health care users. In fact the importance attached to service attributes shows a rather similar pattern across the six countries despite of the differences in health care systems, gaps and bottlenecks in service provision. This suggests common preferences and expectations of health care users across the countries. This assumption requires further testing, however. This could be done by distinguishing the between country variation in satisfaction from the within country variation, e.g. by applying multilevel models.

Other limitations of our study are: we measured satisfaction among health care users only; the limited aspects of service satisfaction studied; the possible cross-country differences in the meaning of satisfaction with quality and access; a three point scale for satisfaction measurement which can be seen as skewed towards the positive end, as well as a ceiling effect, i.e. users may tend to respond positively in a face-to-face interviews; and the relatively small sub-samples of service users per service group analyzed. The low response rate of the survey in some countries can be seen as a limitation. Still irrespective of the non-responses, the procedure applied for the selection of respondents, results in samples representative for the countries indicated by the comparability between the ample characteristics and the countries’ national statistics mentioned in the Results section.

Additionally, the concept of informal patient payments required special attention in terms of the potential sensitiveness of the topic and an adequate mode of data collection. We applied face-to-face interviews as the mode of data collection because the self-administrated mode did not work well during the pre-test. Moreover, the experience with informal patient payments was shared openly in the pre-test without any confusion. We also acknowledge that the combination of high cost and low quality of services in the countries that we studied can be one of the causes of foregoing health care services. We however did not capture the opinion of those who did not use any services. In view of this the satisfaction levels that we report are a user-based indicator as discussed above. We also did not study the possible partial effects of the explanatory variables in the regression analysis. To study such effects, multilevel modeling can be applied.

After acknowledging these limitations, our study provides comparative insights in the satisfaction of health care users with the quality of and the access to health care services in Central and Eastern European countries. As discussed above, the results point to problems in the health care systems seen through the perspective of health care users [70].

## Conclusions

These multi-country findings provide information for health policy making in Central and Eastern European countries. Although the average satisfaction per country is relatively high, the results suggest that there is ample room for improvement. Specifically, many service-users still report to be dissatisfied especially those who pay informally and those unable to pay. The high shares of informal payments and inability of users to deal with the health expenditures lead to doubts about the fairness of the health care provision in Central and Eastern Europe. There is an urgent need to not only acknowledge but also to effectively address this problem. When accessibility and quality of care become key policy goals, and health care governance is improved to be able to deal with informal patient payments, satisfaction of health care users can be improved. In view of this, the satisfaction of health care users with the quality of and access to services can be a useful indicator of health care system performance. The results of this study on satisfaction, informal payments and ability to pay are especially important and relevant for the assessment of health care responsiveness and for the further improvement of the system of service provision. They are also indicative for the understanding of the behavior of health care users that is linked to compliance with the treatment and maintaining the relationship with the care provider.

## Abbreviation

GP, General practitioners

## Additional files

Additional file 1: Wording of the questions used in the study. (DOCX 13 kb) Additional file 2: Socio-demographic characteristics of the sample and sub-samples. (DOCX 47 kb)
