# Supplementary material for: Overall satisfaction of health care users with the quality of and access to health care services: a cross-sectional study in six Central and Eastern European countries
Source: BMC Health Serv Res. 2016 Aug 2;16:342. doi: 10.1186/s12913-016-1585-1 (PMC4971706; doi:10.1186/s12913-016-1585-1)
Supplement: Additional file 1: — Wording of the questions used in the study. (DOCX 13 kb) [file 12913_2016_1585_MOESM1_ESM.docx]

**Additional file 1:**

**Wording of the questions used in the study**

|  | **Consumption and expenditures on outpatient services** |
| --- | --- |
| Q.1 | During the preceding 12 months, how many times did YOU PERSONALLY visit a physician or a physician visited you personally at your home, including any physician in both the public and private system? (Homeopaths and traditional healers who are not physicians, and also dentists are excluded.) [number of visits; 0=none] |
| Q.2 | Considering all types of official and informal cash payments, and in-kind gifts, how much IN TOTAL did you spend (out-of-pocket) on these visits EXCLUDING payments for travelling, transportation by ambulance and pharmaceuticals? [amount in local currency] |
| Q.3 | How much of this amount approximately was for INFORMAL cash payments and in-kind gifts? [amount in local currency] |
| Q.4 | Was it necessary to TAKE OR BORROW cash from family, friends, bank, via credit card or sell assets to cover these formal and informal payments for PHYSICIAN services that you used? [1=Yes;0= No] |
| Q.5 | Are you overall satisfied with the QUALITY of physician services that you used during the last 12 months - yes, no, somewhat? [2=Yes; 1=Somewhat; 0=No] |
| Q.6 | Are you overall satisfied with the ACCESS to physician services that you used during the last 12 months - yes, no, somewhat?? [2=Yes; 1=Somewhat; 0=No] |
|  | **Consumption and expenditures on inpatient services** |
| Q.7 | During the preceding 12 months, how many times were YOU hospitalized (placed in a hospital), including day surgeries or day treatments? (Re-hospitalization, i.e. repeated hospitalization for the same health problem, should be counted separately as a different hospitalization.) [ number of hospitalizations; 0=none] |
| Q.8 | Considering all types of official and informal cash payments, and in-kind gifts, how much IN TOTAL did you spend (out-of-pocket) on these hospitalizations EXCLUDING payments for travelling, transportation by ambulance and pharmaceuticals? [amount in local currency] |
| Q.9 | How much of this amount approximately was for INFORMAL cash payments and in-kind gifts? [amount in local currency] |
| Q.10 | Was it necessary to TAKE OR BORROW cash from family, friends, bank, via credit card or sell assets to cover these formal and informal payments for HOSPITAL services that you used? [1=Yes;0= No] |
| Q.11 | Are you overall satisfied with the QUALITY of hospital services that you used during the last 12 months - yes, no, somewhat? [2=Yes; 1=Somewhat; 0=No] |
| Q.12 | Are you overall satisfied with the ACCESS to hospital services that you used during the last 12 months - yes, no, somewhat? [2=Yes; 1=Somewhat; 0=No] |
|  | **Importance** |
| Q.13 | The card presents a list of seven characteristics of physician’s services.  Please, arrange these characteristics according to their importance TO YOU in case you need to select a physician for a medical consultation due to a MAJOR HEALTH PROBLEM – this means unfamiliar symptoms that cause discomfort or pain, and prevent you from usual activities.  Please rank with 1 the MOST IMPORTANT characteristics, with 2 the second most important and so on till 7, where 7 means least important. Please, do not repeat numbers |
|  | Medical equipment at the physician's office (modern/outdated)  □ Reputation and skills of the physician (known to be good/ unknown)  □ Condition of the physician's office (good condition – renovated/ bad condition)  □ Attitude of the staff (incl. the physician) (polite/ impolite)  □ Travel time to the physician's office (from patient’s home to the office)  □ Waiting time in front of the physician's office (in the waiting room or in the corridor)  □ Amount of money paid by the patient (size of the visit fee) |
| Q.14 | The card presents a list of seven characteristics of hospital services.  Please, arrange these characteristics according to their importance TO YOU in case you need to select a hospital for yourself for a PLANNED SURGERY, which will require a 5-days hospitalization and the illness is not life-threatening.  Please rank with 1 the MOST IMPORTANT characteristics, with 2 the second most important and so on till 7, where 7 means least important. Please, do not repeat numbers. |
|  | □ Medical equipment at the hospital (modern/outdated)  □ Reputation and skills of the surgeon (known to be good/ unknown)  □ Condition of the hospital interior (good condition – renovated/ bad condition)  □ Attitude of the staff (incl. The physician) (polite/ impolite)  □ Travel time to the hospital (from patient’s home to the hospital)  □ Waiting time for the operation (from the referral to the admission)  □ Amount of money paid by the patient (size of the total hospital fee) |
