# Supplementary material for: Overall satisfaction of health care users with the quality of and access to health care services: a cross-sectional study in six Central and Eastern European countries
Source: BMC Health Serv Res. 2016 Aug 2;16:342. doi: 10.1186/s12913-016-1585-1 (PMC4971706; doi:10.1186/s12913-016-1585-1)
Supplement: Additional file 2: — Socio-demographic characteristics of the sample and sub-samples. (DOCX 47 kb) [file 12913_2016_1585_MOESM2_ESM.docx]

**Additional file 2:**

**Socio-demographic characteristics of the sample and sub-samples**

|  |  |  | | Bulgaria | | Hungary | | Lithuania | | Poland | | | Romania | | | Ukraine | | | | Total | | |
| --- | --- | --- | --- | --- | --- | --- | --- | --- | --- | --- | --- | --- | --- | --- | --- | --- | --- | --- | --- | --- | --- | --- |
|  | **TOTAL SAMPLE** | | | | | | | | | | | | | | | | | | |  | | |
| Age | Years | Median | | 52.0 | | 47.0 | | 45.0 | | 43.0 | | | 49.0 | | | 49.0 | | | | 47.0 | | |
|  |  | Mean (SD) | | 50.5 (17.0) | | 46.3 (17.6) | | 46.4 (16.8) | | 44.1 (16.5) | | | 48.5 (17.2) | | | 48.6 (17.6) | | | | 47.39 (17.2) | | |
|  |  | Valid N | | 1003 | | 1037 | | 1012 | | 1000 | | | 1000 | | | 1000 | | | | 6052 | | |
| Gender | Male [0] | N(%) | | 470 (46.9) | | 481 (46.4) | | 437 (43.2) | | 470 (47.0) | | | 417 (41.7) | | | 415 (41.5) | | | | 2690 (44.4) | | |
|  | Female [1] | N(%) | | 533 (53.1) | | 556 (53.6) | | 575 (56.8) | | 530 (53.0) | | | 583 (58.3) | | | 585 (58.5) | | | | 3362 (55.6) | | |
|  |  | Valid N | | 1003 | | 1037 | | 1012 | | 1000 | | | 1000 | | | 1000 | | | | 6052 | | |
| Place of residence | Village [0] | N(%) | | 744 (74.2) | | 831 (80.1) | | 736 (72.7) | | 770 (77.0) | | 775 (77.5) | | | 643 (64.3) | | | 4499 (74.3) | | | |  |
|  | Town (> 200000)[1] | N(%) | | 259 (25.8) | | 206 (19.9) | | 276 (72.3) | | 230 (23.0) | | 225 (22.5) | | | 357 (35.7) | | | 1553 (25.7) | | | |  |
|  |  | Valid N | | 1003 | | 1037 | | 1012 | | 1000 | | 1000 | | | 1000 | | | 6052 | | | |  |
| Education  ISCED^a^ | ISCED 0 | N(%) | | 4 (0.4) | | 2 (0.2) | | 5 (0.5) | | 5 (0.5) | | | 7 (0.7) | | | 3 (0.3) | | 26 (0.4) | | | |  |
|  | ISCED 1 | N(%) | | 50 (5.0) | | 203(19.6) | | 43 (4.2) | | 83 (8.3) | | | 77 (7.7) | | | 16 (1.6) | | 472 (7.8) | | | |  |
|  | ISCED 2 | N(%) | | 176 (17.5) | | 324 (31.2) | | 80 (7.9) | | 162 (16.2) | | | 147(14.7) | | | 54 (5.4) | | 943 (15.6) | | | |  |
|  | ISCED 3 | N(%) | | 540 (53.8) | | 337 (32.5) | | 434 (42.9) | | 611 (61.1) | | | 507 (50.7) | | | 624 (62.4) | | 3053 (50.4) | | | |  |
|  | ISCED 4 | N(%) | | 48 (4.8) | | 34 (3.3) | | 239(23.6) | | 30 (3.0) | | | 77 (7.7) | | | 60 (6.0) | | 488 (8.1) | | | |  |
|  | ISCED 5+6 | N(%) | | 185 (18.4) | | 137 (13.2) | | 211 (20.8) | | 107 (10.7) | | | 185 (18.5) | | | 234 (24.3) | | 1068 (17.7) | | | |  |
|  |  | Valid N | | 1003 | | 1037 | | 1012 | | 998 | | | 1000 | | | 1000 | | 6050 | | | |  |
| Major health problems confirmed by a physician | No [0] | N(%) | | 519 (52.6) | | 569 (54.9) | | 533(52.7) | | 645 (64.5) | | | 574 (57.4) | | | 540 (54.0) | | 3380 (56.0) | | | |  |
|  | Yes [1] | N(%) | | 467 (47.4) | | 468 (45.1) | | 479 (47.3) | | 355 (35.5) | | | 426 (42.6) | | | 460 (46.0) | | 2655 (44.0) | | | |  |
|  |  | Valid N | | 986 | | 1037 | | 1012 | | 1000 | | | 1000 | | | 1000 | | 6035 | | | |  |
| Number of people in the household | Number of persons | Median | | 3.0 | | 2.0 | | 2.0 | | 3.0 | | | 2.0 | | | 3.0 | | 3.0 | | | |  |
|  |  | Mean (SD) | | 2.9 (1.4) | | 2.7 (1.3) | | 2.6 (1.3) | | 3.1 (1.4) | | | 2.6 (1.4) | | | 2.8 (1.4) | | 2.8 (1.4) | | | |  |
|  |  | Valid N | | 1002 | | 1037 | | 1012 | | 996 | | | 999 | | | 1000 | | 6046 | | | |  |
| Net average household income per month [Euro] | Less than 50 Euro | | N (%) | | 11 (1.2) | | 6 (0.6) | | 18 (1.8) | | 1 (0.1) | | | 34 (3.7) | | | 12 (1.3) | | 82 (1.5) | |  |  |
|  | 51 to 75 Euro | | N (%) | | 20 (2.2) | | 5 (0.5) | | 7 (0.7) | | 1 (0.1) | | | 19 (2.1) | | | 23 (2.5) | | 75 (1.3) | |  |  |
|  | 76 to 100 Euro | | N (%) | | 56 (6.2) | | 2 (0.2) | | 22 (2.2) | | 1 (0.1) | | | 29 (3.2) | | | 103 (11.1) | | 213 (3.8) | |  |  |
|  | 101 to 150 Euro | | N (%) | | 98 (10.8) | | 9 (0.9) | | 27 (2.7) | | 11 (1.3) | | | 82 (8.9) | | | 128 (13.7) | | 355 (6.3) | |  |  |
|  | 151 to 200 Euro | | N (%) | | 98 (10.8) | | 22 (2.2) | | 71 (7.2) | | 17 (2.0) | | | 101 (11.0) | | | 149 (16.0) | | 458 (8.2) | |  |  |
|  | 201 to 250 Euro | | N (%) | | 83 (9.1) | | 43 (4.3) | | 103 (10.5) | | 31 (3.6) | | | 94 (10.2) | | | 125 (13.4) | | 479 (8.6) | |  |  |
|  | 251 to 300 Euro | | N (%) | | 73 (8.0) | | 85 (8.5) | | 96 (9.8) | | 37 (4.3) | | | 107 (11.6) | | | 95 (10.2) | | 493 (8.8) | |  |  |
|  | 301 to 350 Euro | | N (%) | | 88 (9.7) | | 61 (6.1) | | 71 (7.2) | | 38 (4.5) | | | 99 (10.8) | | | 110 (11.8) | | 467 (8.4) | |  |  |
|  | 351 to 400 Euro | | N (%) | | 62 (6.8) | | 66 (6.6) | | 64 (6.5) | | 41 (4.8) | | | 87 (9.5) | | | 54 (5.8) | | 374 (6.7) | |  |  |
|  | 401 to 450 Euro | | N (%) | | 52 (5.7) | | 68 (6.8) | | 78 (7.9) | | 36 (4.2) | | | 49 (5.3) | | | 45 (4.8) | | 328 (5.9) | |  |  |
|  | 451 to 500 Euro | | N (%) | | 72 (7.9) | | 84 (8.4) | | 44 (4.5) | | 79 (9.3) | | | 65 (7.1) | | | 26 (2.8) | | 370 (6.6) | |  |  |
|  | 501 to 600 Euro | | N (%) | | 77 (8.5) | | 162 (16.2) | | 91 (9.3) | | 87 (10.2) | | | 54 (5.9) | | | 33 (3.5) | | 504 (9.0) | |  |  |
|  | 601 to 750 Euro | | N (%) | | 60 (6.6) | | 159 (15.9) | | 95 (9.7) | | 144 (16.9) | | | 47 (5.1) | | | 18 (1.9) | | 523 (9.4) | |  |  |
|  | 751 to 1000 Euro | | N (%) | | 46 (5.1) | | 145 (14.5) | | 95 (9.7) | | 165 (19.4) | | | 28 (3.0) | | | 7 (0.8) | | 486 (8.7) | |  |  |
|  | 1001 to 1500 Euro | | N (%) | | 7 (0.8) | | 65 (6.5) | | 67 (6.8) | | 115 (13.5) | | | 16 (1.7) | | | 2 (0.2) | | 272 (4.9) | |  |  |
|  | 1501 to 2000 Euro | | N (%) | | 4 (0.4) | | 13 (1.3) | | 20 (2.0) | | 41 (4.8) | | | 3 (0.3) | | | 1 (0.1) | | 82 (1.5) | |  |  |
|  | 2001 to 3000 Euro | | N (%) | | 0 (0.0) | | 1 (0.1) | | 12 (1.2) | | 7 (0.8) | | | 3 (0.3) | | | 0 (0.0) | | 23 (0.4) | |  |  |
|  | More than 3000 Euro | | N (%) | | 1 (0.1) | | 1 (0.1) | | 2 (0.2) | | 0 (0.0) | | | 3 (0.3) | | | 0 (0.0) | | 7 (0.1) | |  |  |
|  |  | | Valid N | | 908 | | 997 | | 983 | | 852 | | | 920 | | | 931 | | 5591 | |  |  |
| Note: The preliminary analysis showed that the socio-demographic characteristics of the samples are overall comparable to the official statistics in the countries for adults (age 18+ years).  ^a^ ISCED - international standard classification of education, UNESCO | | | | | | | | | | | | | | | | | | |  | |  |  |

|  |  |  | Bulgaria | Hungary | Lithuania | Poland | | Romania | | Ukraine | | Total | | | |
| --- | --- | --- | --- | --- | --- | --- | --- | --- | --- | --- | --- | --- | --- | --- | --- |
|  | **SUB-SAMPLE OF OUT-PATIENT HEALTH CARE USERS** | | | | | | | | | |  | | |  |  |
| Age | Years | Median | 54.0 | 48.0 | 48.0 | 48.0 | | 53.0 | | 50.0 | | 50.0 | | | |
|  |  | Mean (SD) | 52.3 (16.9) | 47.5 (17.8) | 47.6 (17.1) | 46.2 (16.9) | | 51.1 (17.2) | | 50.3 (18.2) | | 49.1 (17.5) | | | |
|  |  | Valid N | 736 | 826 | 739 | 735 | | 651 | | 572 | | 4259 | | | |
| Gender | Male [0] | N(%) | 326 (44.3) | 352 (42.6) | 280 (37.9) | 317 (43.1) | | 231 (35.5) | | 200 (35.0) | | 1706 (40.1) | | | |
|  | Female [1] | N(%) | 410 (55.7) | 474 (57.4) | 459 (62.1) | 418 (56.9) | | 420 (64.5) | | 372 (65.0) | | 2553 (59.9) | | | |
|  |  | Valid N | 736 | 826 | 739 | 735 | | 651 | | 572 | | 4259 | | | |
| Place of residence | Rural area [0] | N(%) | 547 (74.3) | 676 (81.8) | 528 (71.4) | 557 (75.8) | 495 (64.5) | | 369 (64.5) | | 3172 (74.5) | | |  |  |
|  | Town (>200000)[1] | N(%) | 189 (25.7) | 150 (18.2) | 280 (37.9) | 178 (24.2) | 231 (35.5) | | 203 (35.5) | | 1087 (25.5) | | |  |  |
|  |  | Valid N | 736 | 826 | 739 | 735 | 651 | | 572 | | 4259 | | |  |  |
| Education  ISCED^a^ | ISCED 0 | N(%) | 1 (0.1) | 2 (0.2) | 4 (0.5) | 5 (0.7) | 3 (0.5) | | 2 (0.3) | | 17 (0.4) | | |  |  |
|  | ISCED 1 | N(%) | 35 (4.8) | 179 (21.7) | 35 (4.7) | 77 (10.5) | 59 (9.1) | | 11 (1.9) | | 396 (9.3) | | |  |  |
|  | ISCED 2 | N(%) | 133 (18.1) | 256 (31.0) | 52 (7.0) | 119 (16.2) | 102 (15.7) | | 37 (6.5) | | 699 (16.4) | | |  |  |
|  | ISCED 3 | N(%) | 398 (54.1) | 258 (31.2) | 309 (41.8) | 427 (58.3) | 307 (47.2) | | 341 (59.6) | | 2040 (47.9) | | |  |  |
|  | ISCED 4 | N(%) | 35 (4.8) | 24 (2.9) | 174 (23.5) | 23 (3.1) | 56 (8.6) | | 33 (5.8) | | 345 (8.1) | | |  |  |
|  | ISCED 5+6 | N(%) | 134 (18.2) | 107 (13.0) | 165 (22.3) | 82 (11.2) | 124 (19.0) | | 148 (25.9) | | 760 (17.9) | | |  |  |
|  |  | Valid N | 736 | 826 | 739 | 733 | 651 | | 572 | | 4257 | | |  |  |
| Major health problems confirmed by a physician | No [0] | N(%) | 306 (42.4) | 385(46.6) | 324 (43.8) | 398 (54.1) | 282 (43.3) | | 233 (40.7) | | 1928 (45.4) | | |  |  |
|  | Yes [1] | N(%) | 415 (57.6) | 441 (53.4) | 415 (56.2) | 337 (45.9) | 369 (56.7) | | 339 (59.3) | | 2316 (54.6) | | |  |  |
|  |  | Valid N | 736 | 826 | 739 | 735 | 651 | | 572 | | 4259 | | |  |  |
| Number of people in the household | Number of persons | Median | 3.0 | 2.0 | 2.0 | 3.0 | 2.0 | | 3.0 | | 2.0 | | |  |  |
|  |  | Mean (SD) | 2.8 (1.4) | 2.6 (1.3) | 2.5 (1.2) | 3.0 (1.4) | 2.5 (1.2) | | 2.8 (1.3) | | 2.7 (1.3) | | |  |  |
|  |  | Valid N | 736 | 826 | 739 | 732 | 651 | | 572 | | 4256 | | |  |  |
| Net average household income per month [Euro] | Less than 50 Euro | N (%) | 1 (0.1) | 5 (0.6) | 13 (1.8) | 0 (0) | 9 (1.5) | | 6 (1.1) | | 34 (0.9) | | |  |  |
|  | 51 to 75 Euro | N (%) | 14 (2.1) | 4 (0.5) | 2 (0.3) | 0 (0) | 11 (1.8) | | 14 (2.6) | | 45.(1.1) | | |  |  |
|  | 76 to 100 Euro | N (%) | 48 (7.1) | 1 (0.1) | 15 (2.1) | 1 (0.2) | 22 (3.7) | | 69 (13.0) | | 156 (3.9) | | |  |  |
|  | 101 to 150 Euro | N (%) | 67 (10.0) | 6 (0.8) | 15 (2.1) | 10 (1.5) | 58 (9.7) | | 73 (13.8) | | 229 (5.8) | | |  |  |
|  | 151 to 200 Euro | N (%) | 76 (11.3) | 17 (2.1) | 49 (6.8) | 15 (2.3) | 66 (11.0) | | 98 (18.5) | | 321 (8.1) | | |  |  |
|  | 201 to 250 Euro | N (%) | 64 (9.5) | 41 (5.1) | 75 (10.4) | 28 (4.3) | 65 (10.8) | | 71 (13.4) | | 344 (8.7) | | |  |  |
|  | 251 to 300 Euro | N (%) | 52 (7.7) | 71 (8.9) | 71 (9.8) | 32 (4.9) | 70 (11.6) | | 52 (9.8) | | 348 (8.8) | | |  |  |
|  | 301 to 350 Euro | N (%) | 68 (10.1) | 54 (6.8) | 52 (7.2) | 32 (4.9) | 63 (10.5) | | 58 (11.0) | | 327 (8.2) | | |  |  |
|  | 351 to 400 Euro | N (%) | 48 (7.1)) | 55 (6.9) | 49 (6.8) | 31 (4.8) | 63 (10.5) | | 28 (5.3) | | 274 (6.9) | | |  |  |
|  | 401 to 450 Euro | N (%) | 39 (5.8) | 58 (7.3) | 63 (8.7) | 24 (3.7) | 34 (5.7) | | 23 (4.3) | | 241 (6.1) | | |  |  |
|  | 451 to 500 Euro | N (%) | 52 (7.7) | 59 (7.4) | 34 (4.7) | 65 (10.0) | 44 (7.3) | | 12 (2.3) | | 266 (6.7) | | |  |  |
|  | 501 to 600 Euro | N (%) | 56 (8.3) | 129 (16.2) | 65 (9.0) | 66 (10.2) | 35 (5.8) | | 13 (2.5) | | 364 (9.2) | | |  |  |
|  | 601 to 750 Euro | N (%) | 44 (6.5) | 125 (15.7) | 66 (9.1) | 114 (17.6) | 32 (5.3) | | 5 (0.9) | | 386 (9.7) | | |  |  |
|  | 751 to 1000 Euro | N (%) | 34 (5.1) | 110 (13.8) | 70 (9.7) | 119 (18.4) | 15 (2.5) | | 5 (0.9) | | 353 (8.9) | | |  |  |
|  | 1001 to 1500 Euro | N (%) | 6 (0.9) | 51 (6.4) | 54 (7.5) | 75 (11.6) | 10 (1.7) | | 1 (0.2) | | 197 (5.0) | | |  |  |
|  | 1501 to 2000 Euro | N (%) | 3 (0.4) | 10 (1.3) | 17 (2.4) | 29 (4.5) | 2 (0.3) | | 1 (0.2) | | 62 (1.6) | | |  |  |
|  | 2001 to 3000 Euro | N (%) | 0 (0) | 1 (0.1) | 11 (1.5) | 6 (0.9) | 2 (0.3) | | 0 (0.0) | | 20 (0.5) | | |  |  |
|  | More than 3000 Euro | N (%) | 1 (0.1) | 1 (0.1) | 2 (0.3) | 0 (0) | 0 (0) | | 0 (0.0) | | 4 (0.1) | | |  |  |
|  |  | Valid N | 673 | 798 | 723 | 647 | 601 | | 529 | | 3971 | | |  |  |
| ^a^ ISCED - international standard classification of education, UNESCO | | | | | | | | | | | | |  | |  |

|  |  |  | | Bulgaria | | Hungary | | Lithuania | | Poland | | | Romania | | | Ukraine | | | Total | | | |
| --- | --- | --- | --- | --- | --- | --- | --- | --- | --- | --- | --- | --- | --- | --- | --- | --- | --- | --- | --- | --- | --- | --- |
|  | **SUB-SAMPLE OF IN-PATIENT HEALTH CARE USERS** | | | | | | | | | | | | | | | |  | | | |  |  |
| Age | Years | Median | | 58.0 | | 46.0 | | 49.0 | | 56.0 | | | 58.0 | | | 56.0 | | | 54.0 | | | |
|  |  | Mean (SD) | | 56.2 (15.6) | | 47.2 (18.8) | | 49.4 (16.6) | | 50.8 (17.2) | | | 54.2 (17.3) | | | 54.1 (18.5) | | | 51.9 (17.7) | | | |
|  |  | Valid N | | 171 | | 219 | | 165 | | 159 | | | 192 | | | 184 | | | 1090 | | | |
| Gender | Male [0] | N(%) | | 77 (45.0) | | 89 (40.6) | | 61 (37.0) | | 72 (45.3) | | | 75 (39.1) | | | 72 (39.1) | | | 446 (40.9) | | | |
|  | Female [1] | N(%) | | 94 (55.0) | | 130 (59.4) | | 104 (63.0) | | 87 (54.7) | | | 117 (60.9) | | | 112 (60.9) | | | 644 (59.1) | | | |
|  |  | Valid N | | 171 | | 219 | | 165 | | 159 | | | 192 | | | 184 | | | 1090 | | | |
| Place of residence | Village [0] | N(%) | | 142 (83.0) | | 175 (79.9) | | 122 (73.9) | | 128 (80.5) | | 158 (82.3) | | | 128 (69.6) | | | 853 (78.3) | | | |  |
|  | Town (< 200000)[1] | N(%) | | 29 (17.0) | | 44 (20.1) | | 43 (26.1) | | 31 (19.5) | | 34 (17.7) | | | 56 (30.4) | | | 237 (21.7) | | | |  |
|  |  | Valid N | | 171 | | 219 | | 165 | | 159 | | 192 | | | 184 | | | 1090 | | | |  |
| Education  ISCED^a^ | ISCED 0 | N(%) | 2 (1.2) | | 1 (0.5) | | 0 (0) | | 2 (1.3) | | 1 (0.5) | | | 0 (0) | | | 6 (0.6) | | | |  |  |
|  | ISCED 1 | N(%) | 13 (7.6) | | 61 (27.9) | | 3 (1.8) | | 25 (15.7) | | 25 (13.0) | | | 6 (3.3) | | | 133 (12.2) | | | |  |  |
|  | ISCED 2 | N(%) | 39 (22.8) | | 66 (30.1) | | 10 (6.1) | | 28 (17.6) | | 37 (19.3) | | | 14 (7.6) | | | 194 (17.8) | | | |  |  |
|  | ISCED 3 | N(%) | 85 (49.7) | | 63 (28.8) | | 79 (47.9) | | 82 (51.6) | | 88 (45.8) | | | 113 (61.4) | | | 510 (46.8) | | | |  |  |
|  | ISCED 4 | N(%) | 5 (2.9) | | 7 (3.2) | | 39 (23.6) | | 5 (3.1) | | 18 (9.4) | | | 10 (5.4) | | | 84 (7.7) | | | |  |  |
|  | ISCED 5+6 | N(%) | 27 (15.8) | | 21 (9.6) | | 34 (20.6) | | 17 (10.7) | | 23 (12.0) | | | 41 (22.3) | | | 163 (15.0) | | | |  |  |
|  |  | Valid N | 171 | | 219 | | 165 | | 159 | | 192 | | | 184 | | | 1090 | | | |  |  |
| Major health problems confirmed by a physician | No [0] | N(%) | 34 (20.6) | | 84 (38.4) | | 45 (27.3) | | 50 (31.4) | | 56 (29.2) | | | 37 (20.1) | | | 306 (28.2) | | | |  |  |
|  | Yes [1] | N(%) | 131 (79.4) | | 135 (61.6) | | 120 (72.7) | | 109 (68.6) | | 136 (70.8) | | | 147 (79.9) | | | 778 (71.8) | | | |  |  |
|  |  | Valid N | 165 | | 219 | | 165 | | 159 | | 192 | | | 184 | | | 1084 | | | |  |  |
| Number of people in the household | Number of persons | Median | 2.0 | | 2.0 | | 2.0 | | 3.0 | | 2.0 | | | 2.0 | | | 2.0 | | | |  |  |
|  |  | Mean (SD) | 2.8 (1.4) | | 2.7 (1.4) | | 2.6 (1.4) | | 2.8 (1.5) | | 2.4 (1.2) | | | 2.6 (1.2) | | | 2.6 (1.3) | | | |  |  |
|  |  | Valid N | 171 | | 219 | | 165 | | 157 | | 192 | | | 184 | | | 1088 | | | |  |  |
| Net average household income per month [Euro] | Less than 50 Euro | N (%) | 1 (0.6) | | 2 (0.9) | | 2 (1.2) | | 0 (0) | | 3 (1.7) | | | 3 (1.7) | | | 11 (1.1) | | | |  |  |
|  | 51 to 75 Euro | N (%) | 2 (1.3) | | 1 (0.5) | | 2 (1.2) | | 0 (0) | | 7 (4.0) | | | 4 (2.2) | | | 16 (1.6) | | | |  |  |
|  | 76 to 100 Euro | N (%) | 16 (10.1) | | 0 (0) | | 2 (1.2) | | 0 (0) | | 10 (5.6) | | | 25 (14.0) | | | 53 (5.2) | | | |  |  |
|  | 101 to 150 Euro | N (%) | 13 (8.2) | | 3 (1.4) | | 2 (1.2) | | 3 (2.2) | | 22 (12.4) | | | 27 (15.2) | | | 70 (6.8) | | | |  |  |
|  | 151 to 200 Euro | N (%) | 27 (17.0) | | 4 (1.9) | | 14 (8.6) | | 3 (2.2) | | 16 (9.0) | | | 36 (20.2) | | | 100 (9.7) | | | |  |  |
|  | 201 to 250 Euro | N (%) | 19 (11.9) | | 16 (7.5) | | 19 (11.7) | | 6 (4.4) | | 22 (12.4) | | | 24 (13.5) | | | 106 (10.3) | | | |  |  |
|  | 251 to 300 Euro | N (%) | 15 (9.4) | | 18 (8.4) | | 19 (11.7) | | 15 (10.9) | | 22 (12.4) | | | 17 (9.6) | | | 106 (10.3) | | | |  |  |
|  | 301 to 350 Euro | N (%) | 15 (9.4) | | 15 (7.0) | | 16 (9.8) | | 8 (5.8) | | 15 (8.5) | | | 15 (8.4) | | | 84 (8.2) | | | |  |  |
|  | 351 to 400 Euro | N (%) | 14 (8.8) | | 11 (5.1) | | 8 (4.9) | | 8 (5.8) | | 21 (11.9) | | | 10 (5.6) | | | 72 (7.0) | | | |  |  |
|  | 401 to 450 Euro | N (%) | 10 (6.3) | | 13 (6.1) | | 15 (9.2) | | 5 (3.6) | | 5 (2.8) | | | 8 (4.5) | | | 56 (5.4) | | | |  |  |
|  | 451 to 500 Euro | N (%) | 8 (5.0) | | 21 (9.8) | | 5 (3.1) | | 11 (8.0) | | 14 (7.9) | | | 3 (1.7) | | | 62 (6.0) | | | |  |  |
|  | 501 to 600 Euro | N (%) | 6 (3.8) | | 39 (18.2) | | 14 (8.6) | | 13 (9.5) | | 11 (6.2) | | | 5 (2.8) | | | 88 (8.6) | | | |  |  |
|  | 601 to 750 Euro | N (%) | 6 (3.8) | | 33 (15.4) | | 12 (7.4) | | 23 (16.8) | | 6 (3.4) | | | 1 (0.6) | | | 81 (7.9) | | | |  |  |
|  | 751 to 1000 Euro | N (%) | 6 (3.8) | | 21 (9.8) | | 13 (8.0) | | 21 (15.3) | | 2 (1.1) | | | 0 (0) | | | 63 (6.1) | | | |  |  |
|  | 1001 to 1500 Euro | N (%) | 1 (0.6) | | 13 (6.1) | | 12 (7.4) | | 13 (9.5) | | 1 (0.6) | | | 0 (0) | | | 40 (3.9) | | | |  |  |
|  | 1501 to 2000 Euro | N (%) | 0 (0) | | 4 (1.9) | | 5 (3.1) | | 6 (4.4) | | 0 (0) | | | 0 (0) | | | 15 (1.5) | | | |  |  |
|  | 2001 to 3000 Euro | N (%) | 0 (0) | | 0 (0) | | 2 (1.2) | | 2 (1.5) | | 0 (0) | | | 0 (0) | | | 4 (0.4) | | | |  |  |
|  | More than 3000 Euro | N (%) | 0 (0) | | 0 (0) | | 1 (0.6) | | 0 (0) | | 0 (0) | | | 0 (0) | | | 1 (0.1) | | | |  |  |
|  |  | Valid N | 159 | | 214 | | 163 | | 137 | | 177 | | | 178 | | | 1028 | | | |  |  |
| ^a^ ISCED - international standard classification of education, UNESCO | | | | | | | | | | | | | | | | | | | |  | | |
